# Supplementary material for: MOSR and NDHA Genes Comprising G-Quadruplex as Promising Therapeutic Targets against Mycobacterium tuberculosis: Molecular Recognition by Mitoxantrone Suppresses Replication and Gene Regulation
Source: Genes (Basel). 2023 Apr 26;14(5):978. doi: 10.3390/genes14050978 (PMC10217741; doi:10.3390/genes14050978)
Supplement: Supplementary file 1 [file genes-14-00978-s001.zip › genes-2065699-supplementary.pdf]

SUPPLEMENTARY INFORMATION

MOSR and *NDH*A genes comprising G-quadruplex as promising therapeutic targets against *Mycobacterium tuberculosis*: molecular recognition by mitoxantrone suppresses replication and gene regulation

Arpita Dey[1], Kushi Anand[2], Amit Singh[2], Ramasare Prasad[1], Ritu Barthwal[1]*

[1] Department of Biosciences and Bioengineering, Indian Institute of Technology Roorkee, Roorkee, Uttarakhand 247667, India, [2] Centre for Infectious Disease Research, Indian Institute of Science, Bengaluru, Karnataka 560012, India

*Corresponding author: Professor Ritu Barthwal, Email: ritu.barthwal@bt.iitr.ac.in ; barthwalritu.iitr@gmail.com

TABLE OF CONTENTS

1.EXPERIMENTAL SECTION page no. 2- 6

2. TABLES. page no. 7-11

Table S1: List of oligonucleotide sequences used in biophysical experiments

Table S2: List of template and primer sequences used in Taq polymerase stop assay

Table S3: List of primers used in qRT-PCR experiments

Table S4: Melting temperatures, T_m, of *mosR*/*ndhA* G4 DNA sequences

Table S5-S6: Binding affinity of MTX complexed with *mosR* and *ndhA* G4 DNA from SPR

Table S7: Fluorescence decay data of MTX complexed with *mosR* and *ndhA* G4 DNA

Table S8: Ligand (MTX)-induced thermal stabilization (ΔT_m) of *mosR* and *ndhA* G4 DNA

3. FIGURES page no. 12-22

Figure S1: Circular Dichroism spectra of *mosR*/*ndhA* G4 DNA

Figure S2: Thermal melting profiles of *mosR*/*ndhA* G4 DNA

Figure S3: Binding affinity and Stoichiometry - absorption spectra of MTX-*mosR* complex

Figure S4: Binding affinity and Stoichiometry - absorption spectra of MTX-*ndhA* complex

Figure S5: Binding affinity and Stoichiometry - fluorescence spectra of MTX-*mosR* complex

Figure S6: Binding affinity and Stoichiometry - fluorescence spectra of MTX-*ndhA* complex

Figure S7: Binding affinity - CD spectra of MTX-*mosR* and MTX-*ndhA* complexes

Figure S8: Taq polymerase stop assay of MTX with *mosR*/*ndhA* templates

Figure S9: Viability of MTX in replicating model and Gene expression for *Rv1403c* and *hupB*

1. EXPERIMENTAL SECTION

Chemicals

Chemicals like dipotassium hydrogen phosphate (K_2HPO_4), Potassium chloride (KCl), desalted oligonucleotide sequences, 34-mer d-TGGGCTAGCTCTAGGGGGCAGGGCTTTGACGGGT (*mosR*), 32-mer d-TGGGCCTTGTGGGCCTTGTGGGCCTTGTGGGT (*ndhA*) (Table S1), and Mitoxantrone (MTX) used in this study were purchased from Sigma Chem Co., USA. MTX and oligonucleotides were used without additional purification. For the proper formation of G-quadruplex, 34-mer *mosR* and 32-mer *ndhA* oligonucleotide sequences from *Mycobacterium tuberculosis* were dissolved in 10 mM potassium phosphate buffer (KBEPS) (pH 7.0) containing 100 mM KCl, 10 mM K_2HPO_4, 1 mM EDTA and were heated at 95 °C for 5 min followed by slow cooling overnight at room temperature. The sample was stored at 4 °C and shaken gently at regular intervals to ensure its homogeneity. The stock solution of MTX was prepared at a concentration of 1 mM by dissolving it in KBEPS buffer (pH 7.0). The concentration of DNA sequence and MTX were determined using the molar extinction co-efficient $\varepsilon = 69800\ M^{-1}\ cm^{-1}$ at 256 nm (per strand) and $\varepsilon = 20900\ M^{-1}\ cm^{-1}$ at 659 nm, respectively.

Surface Plasmon Resonance (SPR)

The binding of MTX with *mosR* and *ndhA* G4 DNA sequences under *in-vitro* conditions was confirmed and estimated by SPR technique [1-3] using Biacore T200 optical biosensor system (GE Healthcare, Chicago, IL, USA). G4 DNA samples were immobilized onto a streptavidin-derivatized sensor chip, BIACORE SA (GE Healthcare Life Sciences, Little Chalfont, Buckinghamshire, UK), dissolved in HEPES buffer (0.01 M of HEPES, 3 mM of EDTA, 0.005% P20 surfactant) containing 100 mM of KCl at pH 7.4. Only buffer was passed overflow cells 1 and 3 (no immobilized DNA as a reference) whereas *mosR* and *ndhA* G4 DNA were immobilized in flow cells 4 and 2, respectively with a flow rate of 30 µL/min at 25 °C until a steady-state response was reached. The stock MTX solutions were prepared in Millipore purified water. Various concentrations of MTX (0.03-1.5 µM) prepared in HEPES buffer were passed over respective immobilized DNA cells. The drug solutions were dissociated from the complex after passing the regeneration buffer (1 mM of NaCl and 50 µM of NaOH). The response unit was evaluated after subtracting corresponding flow cell 2/4 (immobilized DNA) from reference flow cell 1/3 (HEPES buffer) to get the response from the bound ligand. Analysis and fitting were done by using the Biacore T200 evaluation software that was available with the instrument to get the binding constant (K_b) and Dissociation constant (K_D).

Absorption spectroscopy

Varying concentrations of *mosR*/*ndhA* G4 DNA were mixed with a fixed concentration of MTX (3 μM) to reach mole equivalent ratios (D/N) of MTX Drug (D) to Nucleic acid quadruplex (N) in the range of 0.31–10. Reaction mixtures were incubated at 25 °C for 10 min. Absorbance spectra of each sample were recorded using a Bio UV–visible spectrophotometer (CARY 100, Varian, U.S.A.) equipped with a thermostatic cell holder and quartz cuvette (path length 1 cm) in the wavelength range of 200–800 nm [1-3]. The intrinsic binding constant is calculated using the following equation:

$$[DNA]/(\varepsilon_a-\varepsilon_f) = [DNA]/(\varepsilon_b-\varepsilon_f) + 1/K_b\,(\varepsilon_b-\varepsilon_f) \quad \ldots..1$$

where [DNA] is the concentration of G4 DNA, K_b is the equilibrium constant for binding, ε_a is the apparent absorption coefficient obtained by calculating the ratio of observed absorbance (Optical Density, O.D./A_{obs}) of MTX-G4 DNA complex to the MTX concentration (A_{obs}/[D]), ε_f is the absorption coefficient of the MTX in its free form, and ε_b refers to the absorption coefficient of the MTX in bound form.

Steady-State Fluorescence

Steady-state fluorescence experiments were accomplished using Fluorolog-3 Spectro-fluorimeter LS55 (Horiba Jobin Yvon Spex®). The samples used in absorption spectra were excited (λ_{ex} = 610 nm) and the emission spectra were recorded in the wavelength range of 640–800 nm at 25 °C [1-3]. The fluorescence quenching constant K_{SV} is evaluated using the following equation:

$$F_0/F = 1 + K_{SV}\,[DNA] \quad \ldots..2$$

where F_0 and F are the fluorescence intensities in the absence and presence of quadruplex DNA at 678 nm, [DNA] = N is the concentration of G4 DNA and K_{SV} is the Stern-Volmer quenching constant. The binding constant K_b and binding stoichiometry (*n*) of the complex (the number of ligands binding of DNA) have been determined using the equation:

$$\log[(F_0-F)/F] = \log K_b + n \log [Q] \quad \ldots..3$$

where quencher concentration [Q] = [DNA], K_b is the binding constant and *n* is the number of ligands binding to DNA. The plot of log [(F_0-F)/F] vs. log [DNA] yield K_b and *n*.

To obtain binding constants for simultaneous binding of two molecules of mitoxantrone ligand to DNA, we tried to fit the data in non-linear curve fitting regression analysis by giving user defined equation written in OriginPro 2018 [4]. In case of 2:1 stoichiometry, where two drug (D) molecules bind to a single DNA (N) molecule, we get a cubic equation instead of a quadratic equation involving two binding constants K_{b1} and K_{b2} which is represented by following equation:

$$F = k_0 * C_f + \left\{\frac{k_1 K_{b_1} C_f + k_2 K_{b_1} K_{b_2} C_f^2}{1 + K_{b_1} C_f + K_{b_1} K_{b_2} C_f^2}\right\} C_{Nt} \quad \ldots\ldots 4$$

where, F is the observed fluorescence intensity, k_0, k_1, k_2 are the proportionality constants, C_f is the concentration of free drug, C_{Nt} is total DNA concentration. Newton's method of minimization was used to iteratively determine the free drug concentration at every data point.

Method of Continuous Variation (Job Plot)

To determine the stoichiometry of a binding event, the total concentration of MTX and G- quadruplex DNA were kept constant (3 μM) but their relative mole fractions were varied. At a constant temperature (25 ± 1 °C) emission intensity at λ_{em} = 678 nm was measured after exciting the reaction mixture at λ_{ex} = 610 nm. The difference in fluorescence intensity $\Delta F = F\text{-}F_0$ of free MTX (F_0) and its complex (F) with G4 DNA was plotted as a function of the mole fraction of MTX [1-3]. The inflection point in the plots, which is indicated by a change in slope, gives the mole fraction of MTX bound to G4 DNA. The binding stoichiometry of MTX was calculated using the equation:

$$n = [(1-\chi_{MTX})/\chi_{MTX}] \quad \text{....5}$$

where n is the number of binding sites and χ_{MTX} is the mole fraction of ligand MTX at intersection points of the two observed slopes.

Time-Resolved Fluorescence

Time-resolved fluorescence measurements were performed using the Fluoro-Cube®-Fluorescence lifetime system, (make HORIBA Jobin YvonSpex®) using quartz cuvette (path length 10 mm) operating in a time-correlated single-photon counting (TCSPC) mode [1-3]. The samples were excited by a fixed-wavelength Nano LED (λ_{ex} = 640 nm) with a pulse duration of < 200 ps. All decay traces were measured using a 2048 channel analyzer at 25 ± 1 °C keeping other parameters as time resolution = 0.2 ns, accuracy = ± 0.5 nm, speed 150 nm/s, TAC range = 100 ns. The data were fitted using a deconvolution method of the instrument response function producing the best chi-square fitting values and errors were given as standard deviation obtained from the fits. Three independent experiments were carried out to check the reproducibility and to obtain the average values of the lifetime for MTX and its complex with G4 DNA.

Circular Dichroism

Circular dichroism (CD) spectra were recorded on an Applied Photophysics (Model Chirascan, UK) spectropolarimeter using a 1 mm path length quartz cell, which is equipped with a programmable temperature-controlled cell holder [1-3]. All CD spectra of samples were recorded in the wavelength range 200–700 nm at 25 ± 1 °C with a 1 nm slit width at a 1 nm interval. For titrations experiments to obtain the D/N ratio in the range of 0.1-5.0, the concentration of *mosR*/ndhA G4 DNA was kept constant (20 μM) whereas the MTX concentration was varied. The generated spectra were plotted after

baseline correction and smoothening using the Savitzky-Golay algorithm provided by Chirascan software. To obtain binding constants for 2:1 stoichiometry, we tried to fit the CD data in non-linear curve fitting regression analysis by giving user defined equation written in OriginPro 2018 [4] using the equation:

$$CD = k_0 * C_f + \left\{\frac{k_1 K_{b_1} C_f + k_2 K_{b_1} K_{b_2} C_f^2}{1 + K_{b_1} C_f + K_{b_1} K_{b_2} C_f^2}\right\} C_{Nt} \quad \text{....... 6}$$

where CD is the observed ellipticity (mdegree), k_0, k_1, k_2 are the proportionality constants, C_f is the concentration of free drug, C_{Nt} is total DNA concentration. Newton's method of minimization was used to iteratively determine the free drug concentration at every data point.

Thermal profiling (T_m) using Circular Dichroism (CD)

Thermal melting profiles were performed with a temperature range from 25–95 °C at the rate of 1°C/min using Jasco J-1500 CD Spectrometer (Jasco Japan) equipped with MCB-100 Mini circulation Bath, Peltier unit- controlled cell holder and a xenon lamp [3]. Melting profiles were measured at 260, 287 nm for *ndhA*/its complexes and at 265, 288 nm for *mosR*/its complexes at an interval of 2 °C and plotted after subtracting the buffer as a baseline. The concentration of DNA and MTX were kept constant at 20 μM and 4 μM, respectively to keep the D/N = 5.0.

Thermal profiling (T_m) using Differential Scanning Calorimetry (DSC)

Thermal transitions from ordered quadruplex (helix) DNA to disordered (strand) state is a key factor to determine excess heat capacity measured by using VP DSC Micro-calorimeter (Microcal Inc. Northampton, MA) [1-3]. The sample was scanned from 25 °C to 120 °C at a scan speed of 60 °C/hour at approximately 34 psi pressure. 100 μM G4 DNA sample prepared in KBEPS buffer (pH 7.0), was used for melting transition of unbound form after repeated scanning of buffer under similar conditions. After incubating MTX with G4 DNA at D/N = 5.0 up to 10-12 hours, samples were scanned to acquire the melting profile of bound forms. Both thermograms of bound and unbound form were analyzed using inbuilt VP Viewer software with Origin 7.0. The 3-state/4-state model of curve fitting was used to fit the raw thermograms of unbound quadruplex DNA and its complexes.

***Taq* polymerase stop assay**

The template DNA strand, containing *mosR* and *ndhA* and all primers were procured from Sigma Aldrich (Table S2). PCR method was performed in a 10 μL reaction containing a concentration of 1 μg/μL template DNA (100 mM KCl), 10 mM primer, 2.5 mM dNTPs, 5 Units of *Taq* polymerase (Thermo Fisher Scientific, USA) and different concentrations of MTX (1.56–50 μM). Reaction mixture was prepared in nuclease-free water. Negative control was considered as no template in the

reaction mixture, producing no product. The PCR based amplification was carried out by using initial denaturation at 95 °C for 5 min, followed by 30 cycles of denaturation at 95 °C for 30 s, annealing and extension at 64 °C for 30 s, and the final extension at 72 °C for 50 s using a thermal cycler (model T100, Bio-Rad, USA); after the end of the reaction, PCR tubes were kept at 4 °C. The amplified PCR products were then separated by agarose gel electrophoresis by mixing with 6x loading dye, resolved on a 3% w/w agarose gel, and image analysis was carried out using Gel Doc EZ imager (Bio-Rad, USA) by staining with ethidium bromide.

Gene expression by qRT-PCR

Wild type *Mycobacterium tuberculosis* (H37Rv) strain was grown in a 7H9 medium supplemented with 1x ADS (Albumin Dextrose Saline) to an OD_{600}-0.4 and exposed to 5X MIC of MTX for 1 h in a rotating incubator (180 rpm) at 37 °C [5,6]. After treatment, total RNA was purified as described. After purification and DNase treatment cDNA was synthesized from 500 ng of total RNA using a script cDNA synthesis kit as per manufacturer instruction. Gene-specific primers for *Mtb mosR* (FP2_KAAD_*Rv1049*RT- 5-'CGAATGCGCTTGCTACACC-3' and RP2_KAAD_*Rv1049*RT- 5-'CCTTCCGACAGCGAGATCAC-3') and *ndhA* FP1_KAAD_*Rv0392*cRT- 5-'AGACGGTCACGTCGAAATTG-3' and RP1_KAAD_*Rv0392*cRT- 5-'GCCGAAGTAGGACTGCTGTG-3') were selected (Table S3) for RT-PCR (Step one Applied Biosystem). iQ™ SYBR Green Supermix was used for gene expression analysis and data was normalized to 16S rRNA expression. The experiment was carried out with a minimum of two biological replicates and fold change was calculated concerning untreated control.

References

1. Ritu Barthwal and Zia Tariq, (2018) Molecular Recognition of Parallel G-quadruplex [d-(TTGGGGT)]4 Containing Tetrahymena Telomeric DNA Sequence by Anticancer Drug Daunomycin: NMR-Based Structure and Thermal Stability, Molecules, **23**, 2266-2292.
2. Zia Tariq and Ritu Barthwal, (2018) Binding of anticancer drug daunomycin to parallel G-quadruplex DNA [d-(TTGGGGT)]4 leads to thermal stabilization: A multi-spectroscopic investigation, International Journal of Biological Macromolecules, **120**, 1965-1974.
3. Arpita Dey, Kumud Pandav, Mala Nath, Ritu Barthwal, Ramasare Prasad, (2022) Molecular recognition of telomere DNA sequence by 2, 6 anthraquinone derivatives leads to thermal stabilization and induces apoptosis in cancer cells, International Journal of Biological Macromolecules **221**, 355-370.
4. Hargrove,A.E., Zhong,Z., Sessler,J.L. and Anslyn,E.V. (2010) Algorithms for the determination of binding constants and enantiomeric excess in complex host: Guest equilibria using optical measurements. *New J. Chem.*, **34**, 348–354.
5. Padiadpu,J., Baloni,P., Anand,K., Munshi,M.H., Thakur,C., Mohan,A., Singh,A. and Chandra,N. (2016) Identifying and tackling emergent vulnerability in drug-resistant mycobacteria. *ACS Infect. Dis.*, **2**, 592-607.
6. Anand,K., Tripathi,A., Shukla,K. and Malhotra,N. (2021) Redox Biology Mycobacterium tuberculosis SufR responds to nitric oxide via its 4Fe – 4S cluster and regulates Fe – S cluster biogenesis for persistence in mice. *Redox Biol.*, **46**, 102062.

2. TABLES

Table S1 List of oligonucleotide sequences for biophysical measurements

Experiment	Sequence
Circular Dichroism, Absorption, Fluorescence, and Differential Scanning Calorimetry	*mosR*-TGGGCTAGCTCTAGGGGGCAGGGCTTTGACGGGT<br>*ndhA*-TGGGCCTTGTGGGCCTTGTGGGCCTTGTGGGT
Surface Plasmon Resonance	*mosR*-5'biotin-TGGGCTAGCTCTAGGGGGCAGGGCTTTGACGGGT<br>*ndhA*-5'biotin-TGGGCCTTGTGGGCCTTGTGGGCCTTGTGGGT

Table S2 List of template and primers sequence used in Taq polymerase stop assay

Template name	Sequence
mosR	TTTT TGGGCTAGCTCTAGGGGGCAGGGCTTTGACGGGT<br>TTTTCTGCATATAAGCAGCTGCTTTTTGCC
ndhA	TTTT TGGGCCTTGTGGGCCTTGTGGGCCTTGTGGGT<br>TTTTCTGCATATAAGCAGCTGCTTTTTGCC
Taq primer	GGCAAAAAGCAGCTGCTTATATGCAG

Table S3 List of primers sequences for *mosR* and *ndhA* gene for processing qRT-PCR

**Gene name**	**Forward primer**	**Reverse primer**
mosR- Rv1049RT	5-'CGAATGCGCTTGCTACACC-3'	5-'CCTTCCGACAGCGAGATCAC-3'
ndhA- Rv0392cRT	5-'AGACGGTCACGTCGAAATTG-3'	5-'GCCGAAGTAGGACTGCTGTG-3'

Table S4A Melting temperatures, T_m, of *mosR* G4 DNA sequences obtained from variation of ellipticity with temperature under different solution conditions: KBPES buffer containing 0, 50, 100, 150 mM KCl and in water.

Sample	T_m (°C) of *mosR*					
0 KCl	29	42	49	60	75	90
50 mM KCl	28	40		61		86
100 mM KCl	31	45		60	75	86
150 mM KCl	35		53		70	85
Water	31	45		65	74	90

Table S4B Melting temperatures, T_m, of *ndhA* G4 DNA sequences obtained from variation of ellipticity with temperature under different solution conditions: KBPES buffer containing 0, 50, 100, 150 mM KCl and in water.

Sample	T_m (°C) of *ndhA*					
0 KCl	24	38	52	62	77	91
50 mM KCl	26	39	56		75	89
100 mM KCl	30	45			73	
150 mM KCl	31	44	55	65	80	92
Water	28	41	54		72	87

Table S5 Surface Plasmon Resonance results showing maximum Response Unit (RU_{max}), association rate constant (k_a), dissociation rate constant (k_d), equilibrium Dissociation constant (K_D) and equilibrium binding constant (K_b) on binding of MTX with *mosR* in the concentration range 0.03-5.0 μM. χ^2 for the goodness of fit are also shown.

Experiment	Flow rate (μl/min)	RU_{max}	k_a ($M^{-1}s^{-1}$)	k_d (s^{-1})	K_D (M)	K_b (M^{-1})	χ^2
Kinetics run 1	30	508.2	$6.216x10^4$	0.1603	$2.58x10^{-6}$	$3.88x10^5$	55
Steady State run 1	30	505.1		-	$3.85 x10^{-6}$	$2.60x10^5$	48
Kinetics run 2	30	469.8	$6.293x10^4$	0.1579	$2.51x10^{-6}$	$3.98 x10^5$	69
Steady State run 2	30	415.7		-	$2.90x10^{-6}$	$3.44 x10^5$	171
Kinetics run 3	30	468.2	$6.339x10^4$	0.1714	$2.71 x10^{-6}$	$3.70 x10^5$	49
Steady State run 3	30	401.4		-	$2.90 x10^{-6}$	$3.44 x10^5$	146
Average from Kinetics data						$3.85 x10^5$	
Average from Steady State data						$3.16 x10^5$	

Table S6 Surface Plasmon Resonance results showing maximum Response Unit (RU_{max}), association rate constant (k_a), dissociation rate constant (k_d), equilibrium Dissociation constant (K_D) and equilibrium binding constant (K_b) on binding of MTX with *ndhA* in the concentration range 0.03-5.0 μM. χ^2 for the goodness of fit are also shown.

Experiment	Flow rate (μl/min)	RU_{max}	k_a ($M^{-1}s^{-1}$)	k_d (s^{-1})	K_D (M)	K_b (M^{-1})	χ^2
Kinetics run 1	30	179.4	$5.870x10^5$	0.0437	$7.44x10^{-8}$	$1.34 x10^7$	89
Steady State run 1	30	401.3		-	$7.29 x10^{-7}$	$1.37x10^6$	176
Kinetics run 2	30	212.3	$2.184x10^7$	1.2960	$5.93 x10^{-8}$	$1.68 x10^7$	71
Steady State run 2	30	476.7		-	$1.38x10^{-6}$	$7.25 x10^5$	129
Kinetics run 3	30	192.2	$7.043x10^5$	0.0877	$1.25x10^{-7}$	$8.03 x10^6$	42
Steady State run 3	30	360.0		-	$7.13x10^{-7}$	$1.40 x10^6$	140
Average from Kinetics data						$1.27x10^7$	
Average from Steady State data						$1.17x10^6$	

Table S7A Fluorescence decay lifetime (τ in ns), their relative proportions (B in percentage) and average lifetime (τ in ns) from fluorescence decay profiles of 3 μM free MTX and its complexes with *mosR* at varying MTX to *mosR* G4 DNA ratio. χ^2/DOF (degrees of freedom) for the goodness of fit are also shown.

Sample	τ1	τ2	B1	B2	Average τ	χ^2/DOF
Free MTX	0.11	0.47	90.42	9.58	0.123	1.1/459
MTX/*mosR* = 0.5	0.21	0.51	59.59	40.41	0.281	1.3/524
MTX/*mosR* = 1.0	0.18	0.46	49.98	50.02	0.258	1.3/501
MTX/*mosR* = 2.0	0.09	0.39	60.98	39.02	0.134	1.1/734
MTX/*mosR* = 4.0	0.11	0.36	79.04	20.96	0.123	1.0/692

Table S7B Fluorescence decay lifetime (τ in ns), their relative proportions (B in percentage) and average lifetime (τ in ns) from fluorescence decay profiles of 3 μM free MTX and its complexes with *ndhA* at varying MTX to *ndhA* G4 DNA ratio. χ^2/DOF (degrees of freedom) for the goodness of fit are also shown.

Sample	τ1	τ2	B1	B2	Average τ	χ^2/DOF
Free MTX	0.11	0.47	90.42	9.58	0.123	1.1/459
MTX/*ndhA* = 0.5	0.18	0.49	59.60	40.40	0.238	1.2/507
MTX/*ndhA* = 1.0	0.18	0.49	64.45	35.35	0.236	1.1/531
MTX/*ndhA* = 2.0	0.14	0.51	100.91	-0.09	0.137	1.1/678

Table S8A Melting temperatures (T_m in ^oC) and subsequent thermal stabilization (ΔT_m in ^oC) due to binding from melting profiles obtained from data of Circular Dichroism at 288/287 nm as a function of temperature for free *mosR and ndhA* and their complex with MTX at D/N = 5.0.

Sample	T_{m1}	ΔT_{m1}	T_{m2}	ΔT_{m2}	T_{m3}	ΔT_{m3}	T_{m4}	ΔTm_4	T_{m5}	ΔT_{m5}
Free *mosR*	25	-	32	-	54	-	68	-	85	-
MTX + *mosR*	25	-	39	7	58	4	79	11	-	-
Free *ndhA*	28	-	39	-	45	-	60	-	84	-
MTX + *ndhA*	28	-	39	-	57	12	80	20	90	6

Table S8B Melting temperatures (T_m in ^oC) and subsequent thermal stabilization (ΔT_m in ^oC) due to binding from melting profiles obtained from data of Circular Dichroism at 265/260 nm as a function of temperature for free *mosR and ndhA* and their complex with MTX at D/N = 5.0.

Sample	T_{m1}	ΔT_{m1}	T_{m2}	ΔT_{m2}	T_{m2}'	$\Delta T_{m2}'$	T_{m3}	ΔT_{m3}	T_{m3}'	$\Delta T_{m3}'$	T_{m4}	ΔT_{m4}	T_{m5}
Free *mosR*	28	-	43	-	-	-	60	-	60	-	74	-	87
MTX+*mosR*	29	1	42, 54	11			73	13	86	26			
Free *ndhA*	26	-	38	-	38		60	-	60	-	85	-	-
MTX+*ndhA*	28	2	40, 45	2, 7	54	16	64	4	73	13	93	8	

3. FIGURES

A

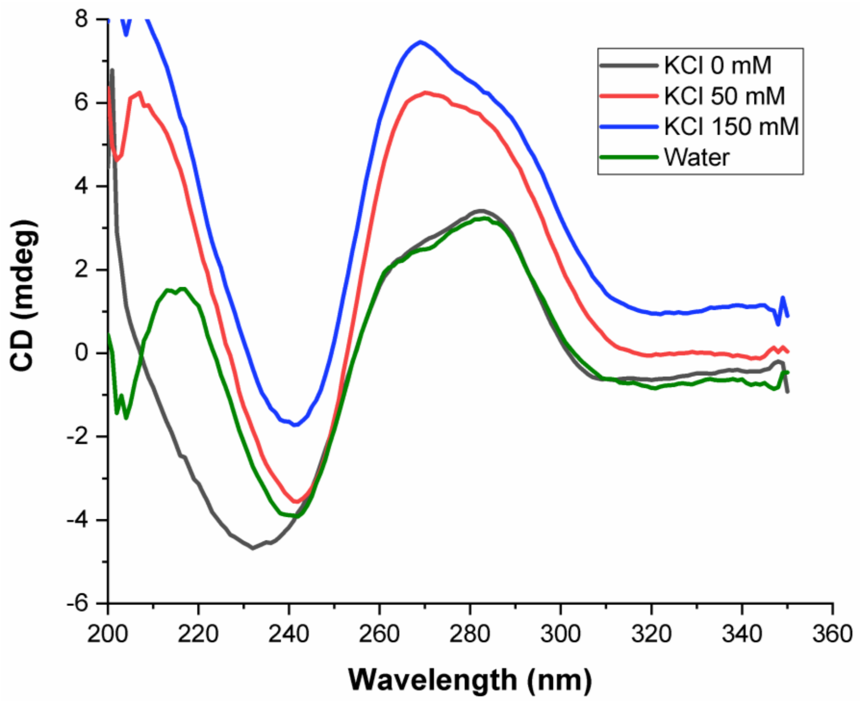

B

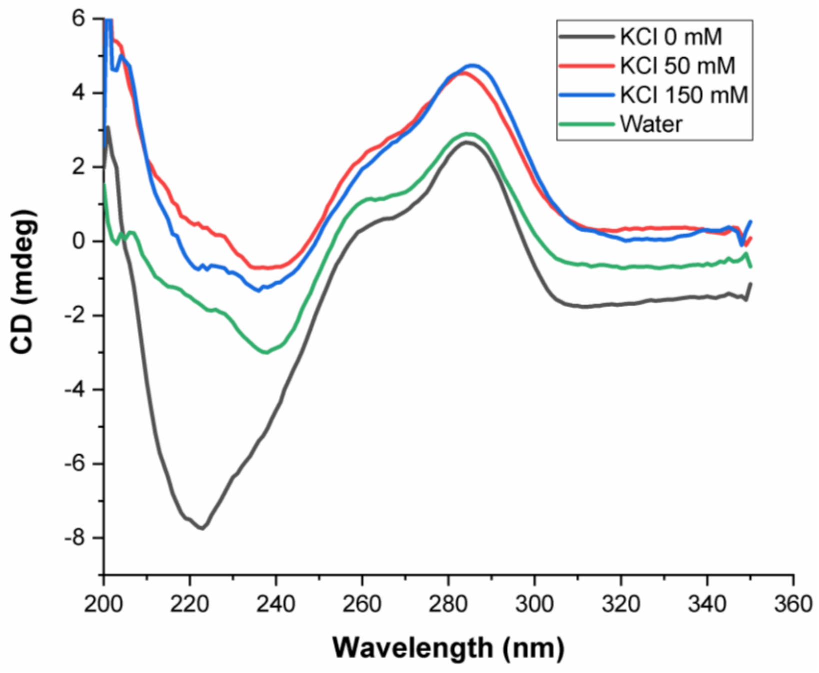

Figure S1 Circular dichroism spectra of G4 DNA: (A) *mosR* and (B) *ndhA* in KBPES buffer (pH 7.0) containing 0, 50,150 mM KCl and in water

mosR

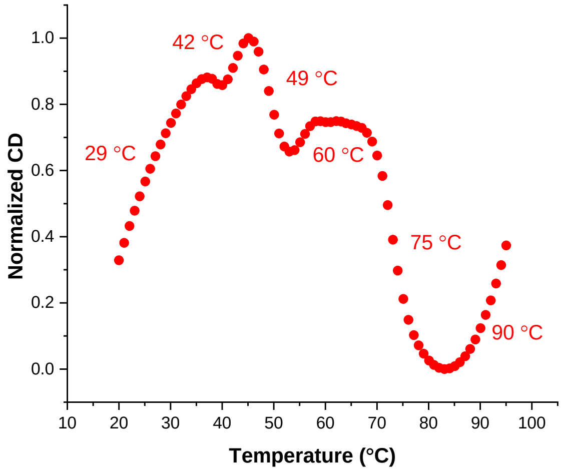

O KCl

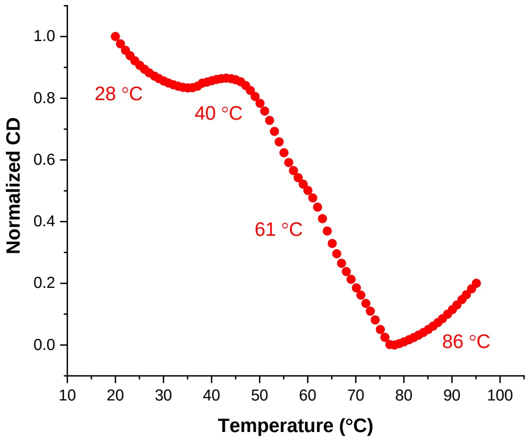

50 KCl

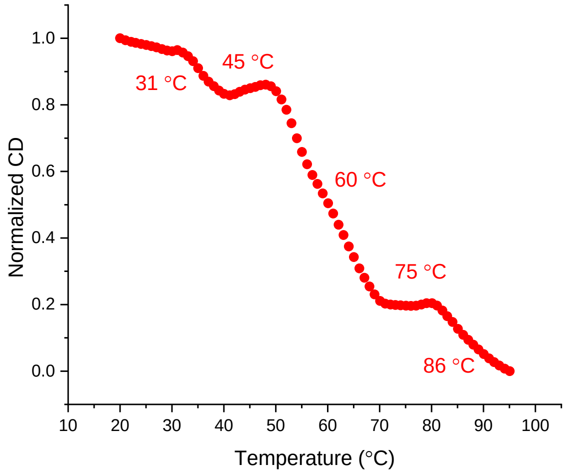

100 KCl

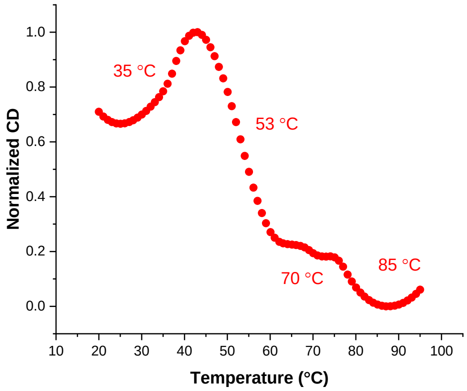

150 KCl

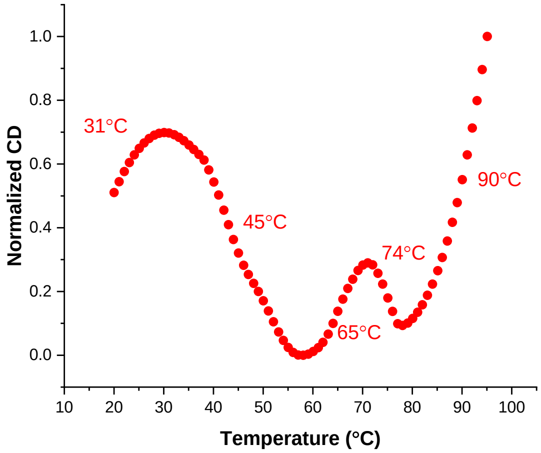

water

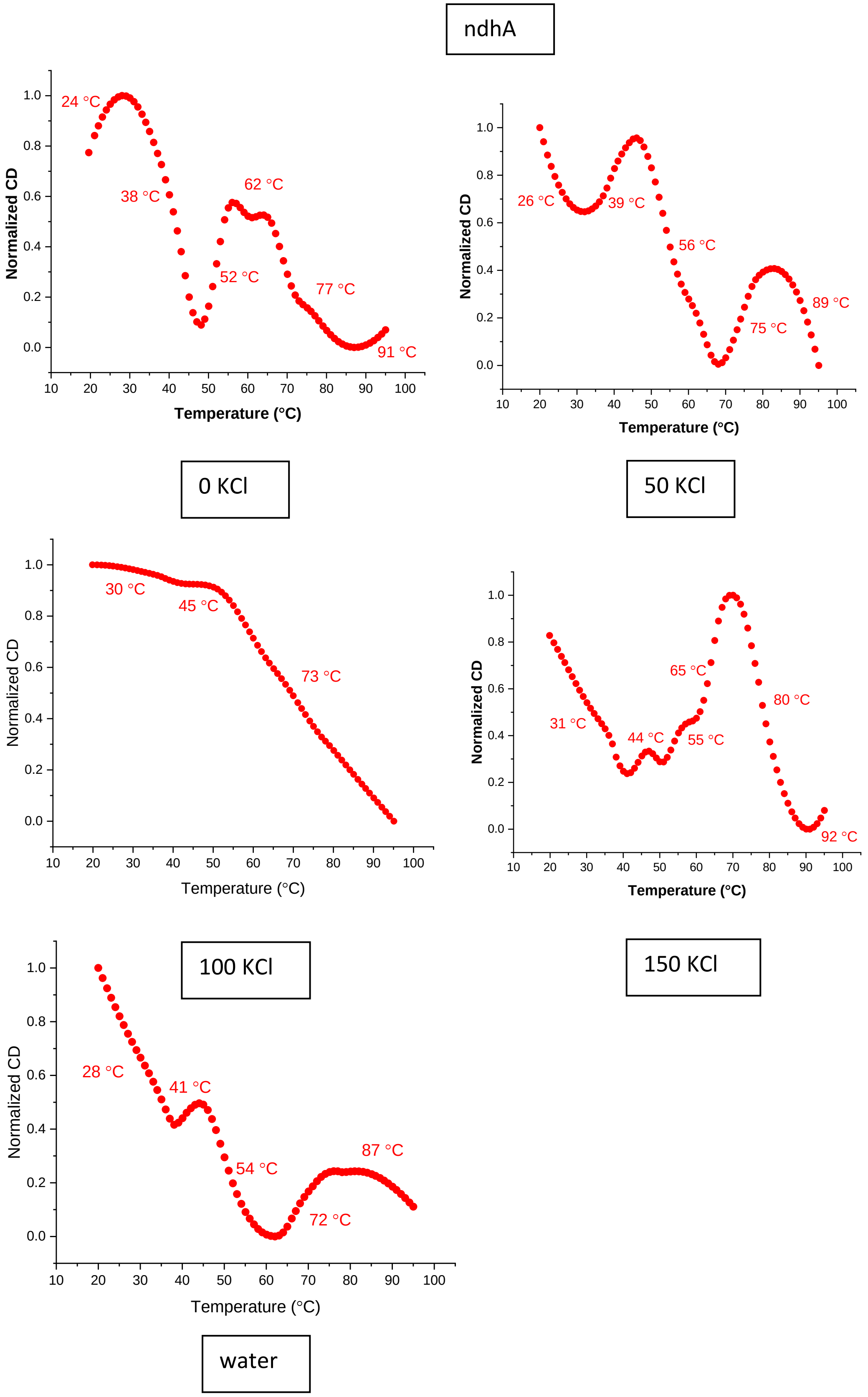

Figure S2 Thermal melting profiles of free *mosR* and *ndhA* G4 DNA in different concentrations of KCl in KBPES buffer and in water.

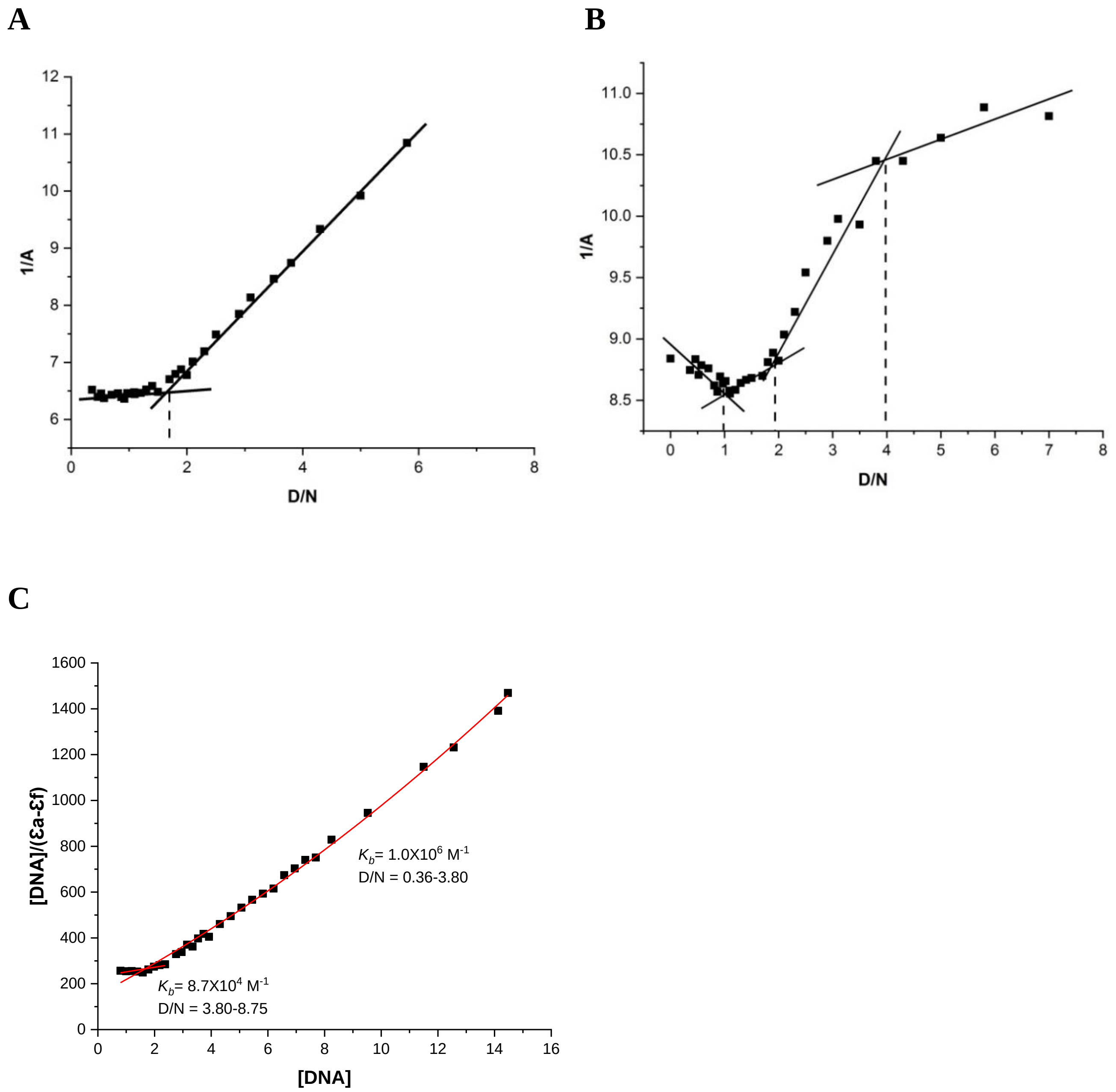

Figure S3 Titration of 7 μM mitoxantrone (MTX) with *mosR* G4 DNA sequence: (A) Plot of reciprocal of absorbance (1/A) as a function of D/N at 624 nm showing inflection at D/N = 1.8 suggesting stoichiometry of 2:1; (B) Plot of reciprocal of absorbance (1/A) as a function of D/N at 678 nm showing inflection at D/N = 1.2, 2.0, and 3.7 suggesting stoichiometry of 1:1, 2:1, and 4:1; (C) Plot of [DNA]/(ε_a-ε_f) as a function of concentration of *mosR*, [DNA], yielding binding constant (K_b).

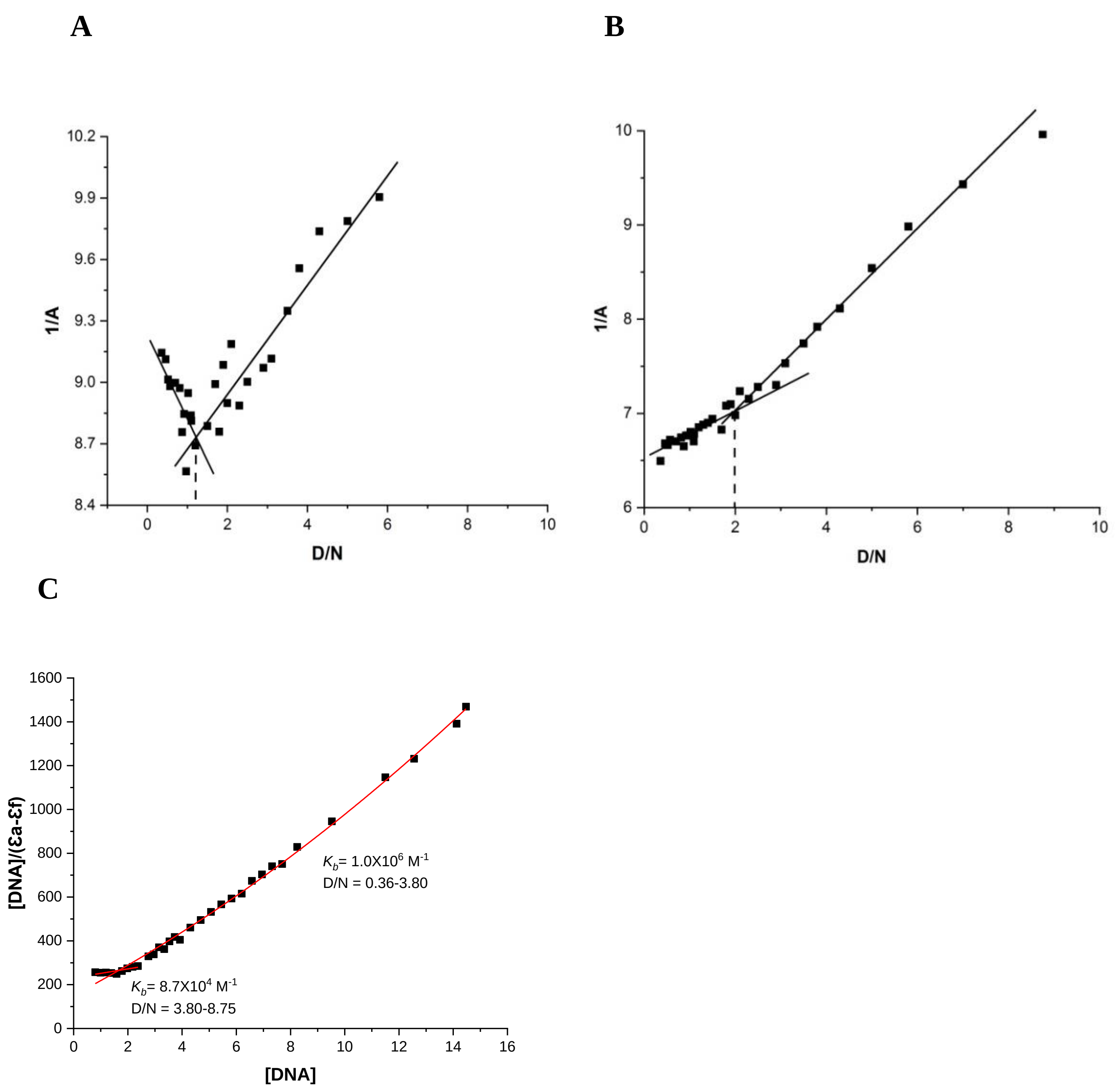

Figure S4 Titration of 7 µM MTX with *ndhA* G4 DNA sequence: (A) Plot of reciprocal of absorbance (1/A) as a function of D/N at 624 nm showing inflection at D/N = 1.2 suggesting stoichiometry of 1:1; (B) Plot of reciprocal of absorbance (1/A) as a function of D/N at 678 nm showing inflection at D/N = 2.0 suggesting stoichiometry of 2:1; (C) Plot of [DNA]/(ε_a-ε_f) as a function of concentration of *ndhA*, [DNA], yielding binding constant (K_b).

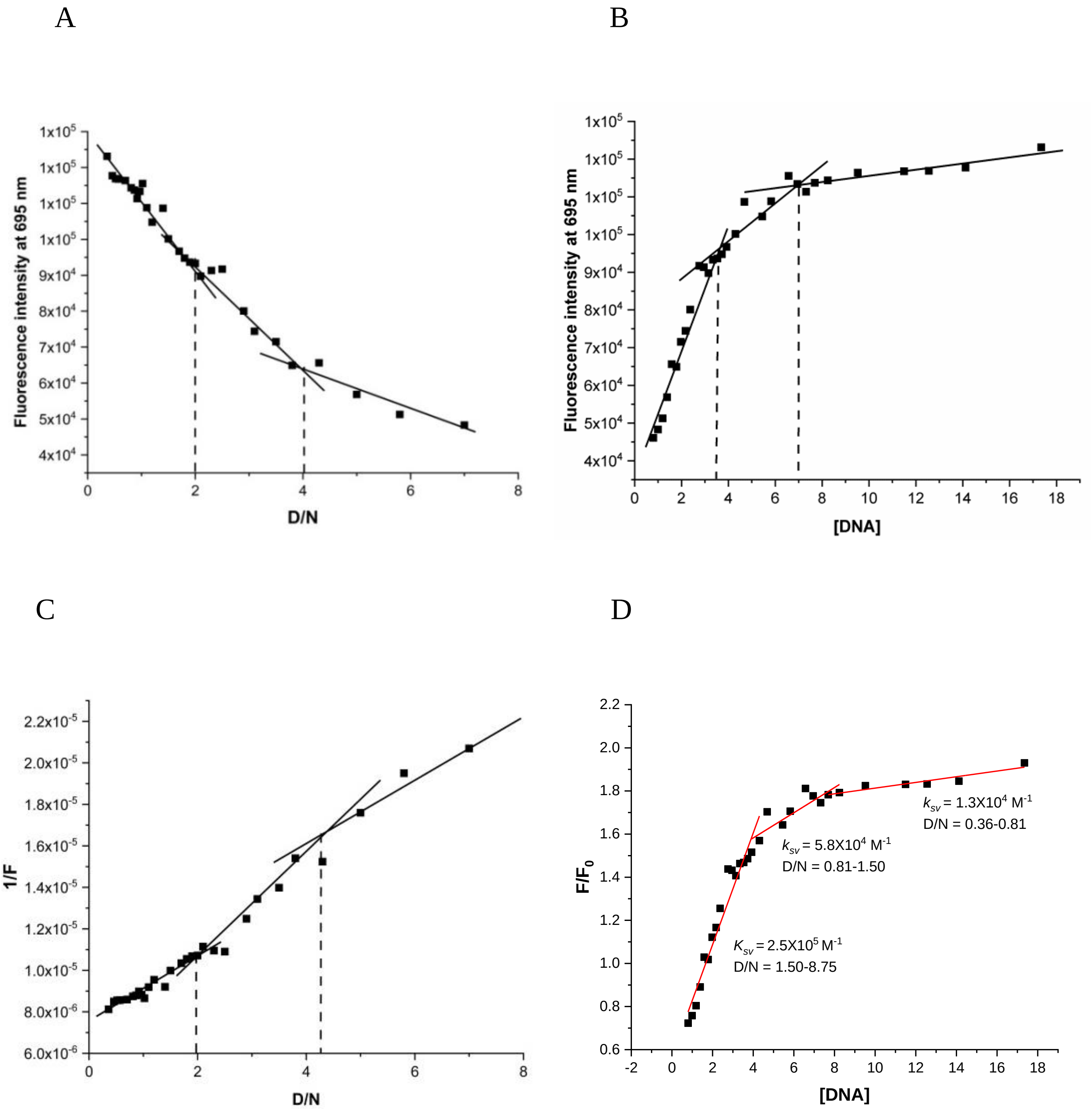
A
Fluorescence intensity at 695 nm
D/N
B
Fluorescence intensity at 695 nm
[DNA]
C
1/F
D/N
D
F/F0
[DNA]
k_{sv} = 1.3X10^4 M^{-1}
D/N = 0.36-0.81
k_{sv} = 5.8X10^4 M^{-1}
D/N = 0.81-1.50
K_{sv} = 2.5X10^5 M^{-1}
D/N = 1.50-8.75

E

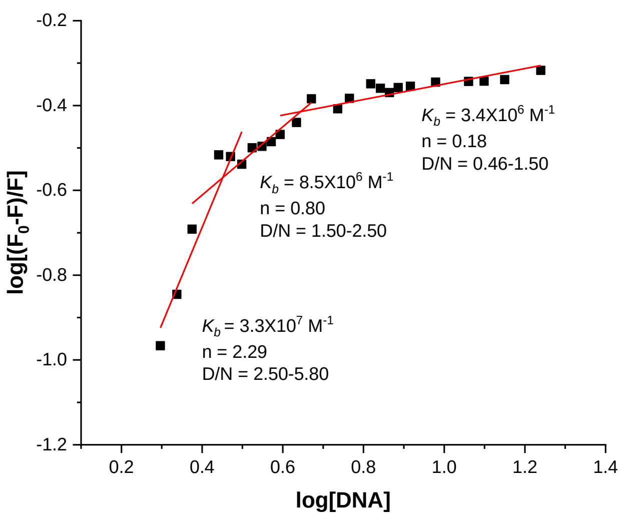

F

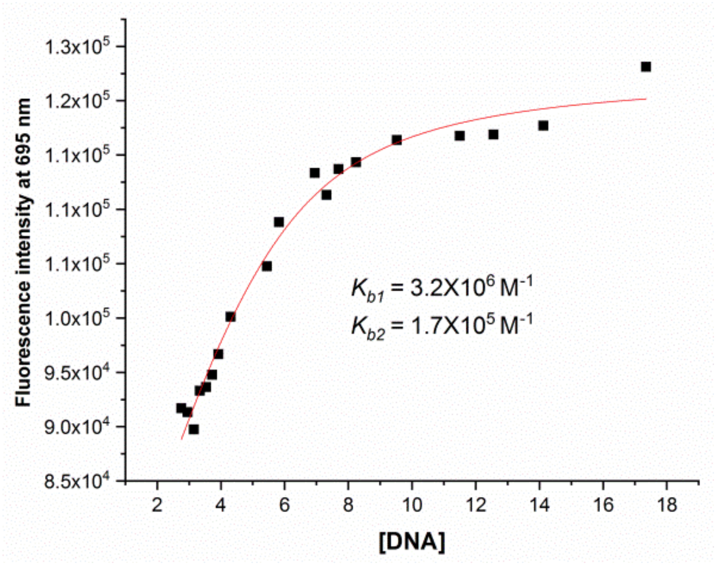

Figure S5 Titration of 7 µM MTX with increasing concentration of *mosR* G4 DNA sequence at D/N = 0.3–8.7 using fluorescence: λ_{ex} = 610 nm and λ_{em} = 695 nm (A) Plot of fluorescence (F) as a function of D/N showing change in slope at D/N = 2.0 and 4.0 suggesting stoichiometry of 2:1 and 4:1; (B) Plot of fluorescence as a function of concentration of *mosR*, [DNA], showing change in slope at [DNA] = 3.5 and 7.0 µM suggesting stoichiometry of 2:1 and 1:1; (C) Plot of reciprocal of fluorescence intensity (1/F) as a function of D/N ratio showing inflection at D/N = 2.0 and 4.2 suggesting stoichiometry of 2:1, and 4:1; (D) Plot of F_0/F versus concentration of [DNA] giving Stern-Volmer Quenching constant (K_{SV}); (E) Plot of log [(F_0-F)/F] versus log [DNA] showing binding constant (K_b) at different ranges of D/N; (F) Nonlinear fit of fluorescence intensity F as a function of [DNA] yielding binding constant (K_b) using Origin software.

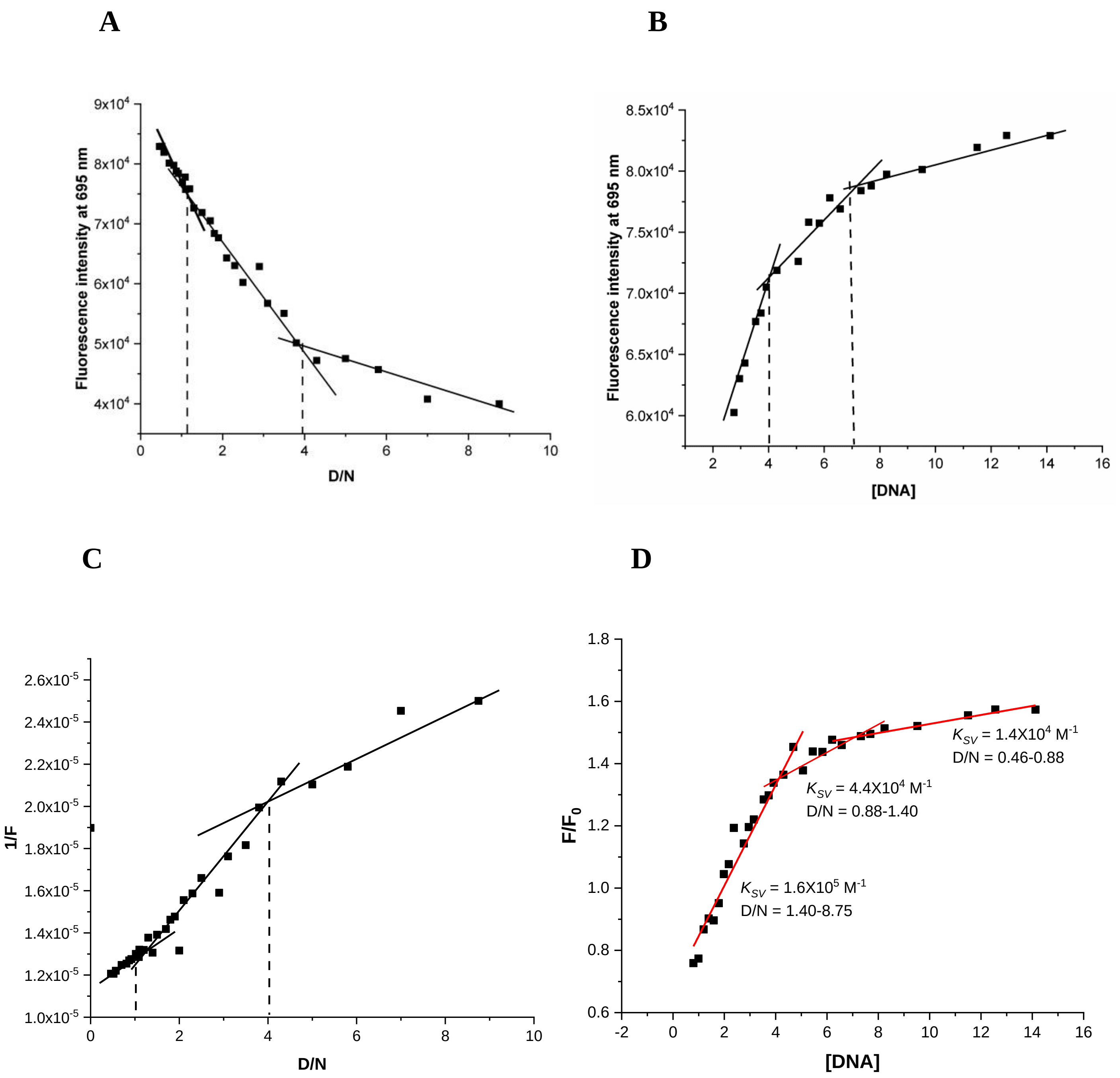
A
Fluorescence intensity at 695 nm
D/N
B
Fluorescence intensity at 695 nm
[DNA]
C
1/F
D/N
D
F/F0
[DNA]
KSV = 1.4X10^4 M^-1
D/N = 0.46-0.88
KSV = 4.4X10^4 M^-1
D/N = 0.88-1.40
KSV = 1.6X10^5 M^-1
D/N = 1.40-8.75

E **F**

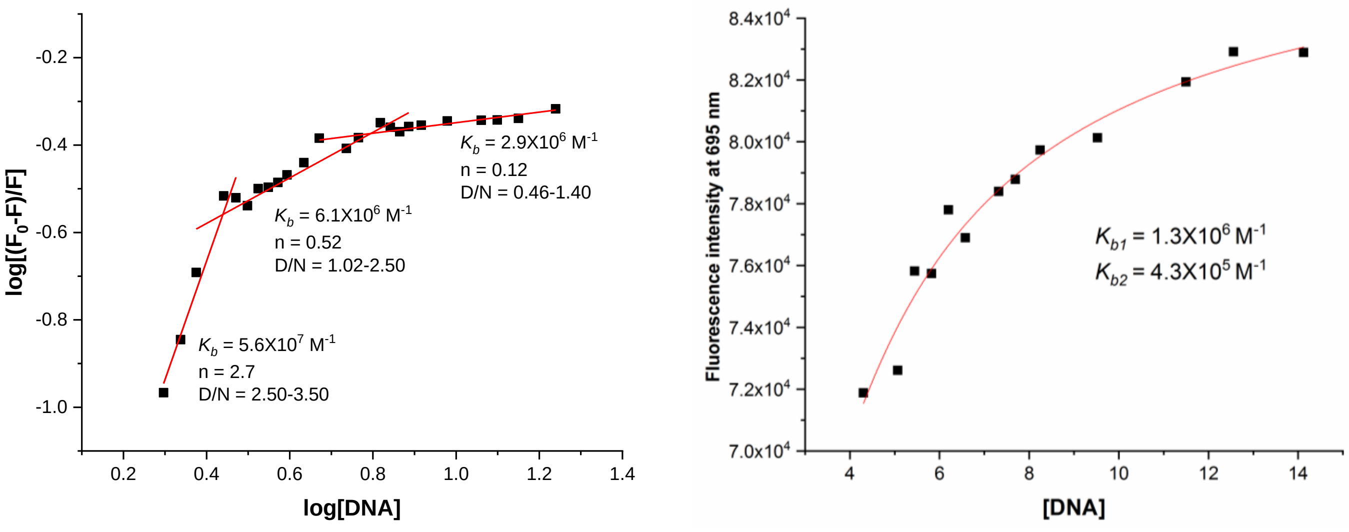

Figure S6 Titration of 7 µM MTX with increasing concentration of *ndhA* G4 DNA sequence at D/N = 0.3–8.7 using fluorescence: λ_{ex} = 610 nm and λ_{em} = 695 nm (A) Plot of fluorescence (F) as a function of D/N showing change in slope at D/N =1.0 and 4.0 suggesting stoichiometry of 1:1 and 4:1; (B) Plot of fluorescence as a function of concentration of *ndhA*, [DNA], showing change in slope at 4.0 and 7.0 µM suggesting stoichiometry of 2:1 and 1:1; (C) Plot of reciprocal of fluorescence intensity (1/F) as a function of D/N ratio showing inflection at D/N = 1.0 and 4.0 suggesting stoichiometry of 1:1 and 4:1; (D) Plot of F_0/F versus [DNA] giving Stern-Volmer Quenching constant (K_{SV}); (E) Plot of log [(F_0-F)/F] versus log [DNA] showing binding constant (K_b) at different ranges of D/N; (F) Nonlinear fit of fluorescence intensity F as a function of [DNA] yielding binding constants (K_b) using Origin software.

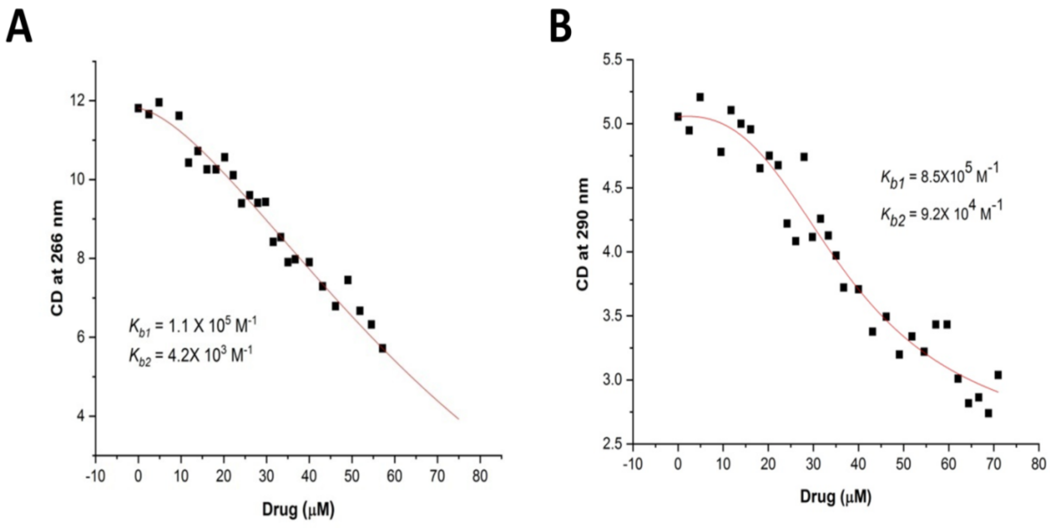

Figure S7 Nonlinear fitted curve (red color) of observed CD (millidegrees) as a function of concentration of MTX drug (μM) for binding with 20 μM (A) *mos*R and (B) *ndhA* G4 DNA sequences at 266 and 290 nm, respectively yielding binding constant (K_b) using Origin software

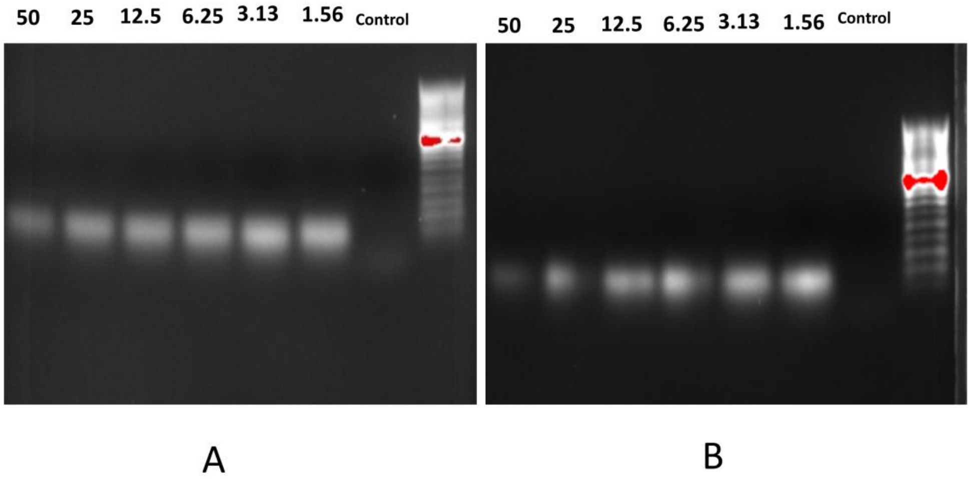

Figure S8 Taq polymerase stop assay after treatment of different concentrations of MTX (50-1.56 μM) with the template of (A) *mosR* and (B) *ndhA*. MTX without any template is taken as control in the experiment.

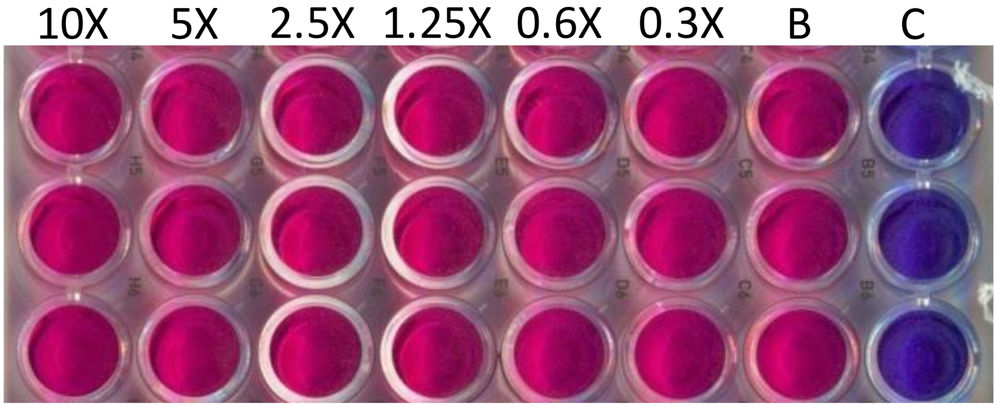

Figure S9a Viability of MTX in replicating model: *Mtb* H37Rv was treated with different concentration of MTX (10X MIC-0.3X MIC). Alamar blue assay was performed to analyze the cytotoxicity of MTX. B - bacterial control without MTX, D - Drug control without bacteria.

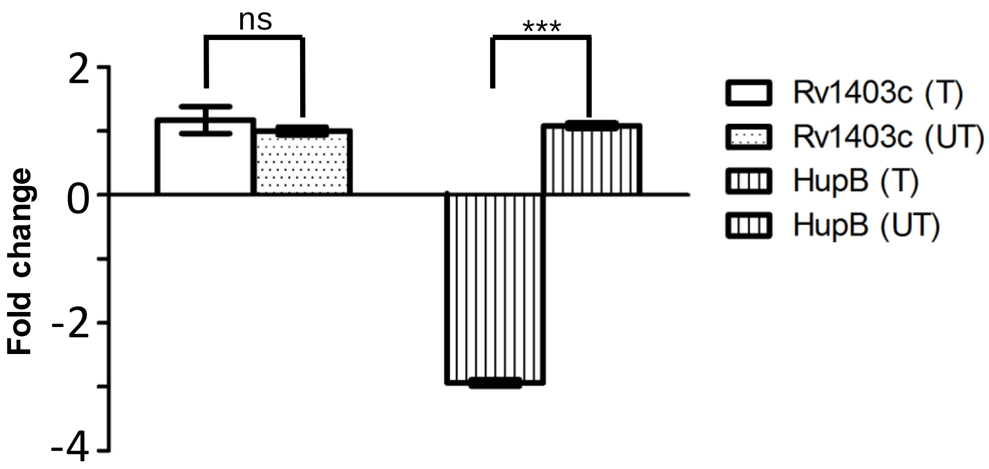

Figure S9b *Mtb* H37Rv was treated with 5XMIC of MTX; RNA was isolated and subjected to qRT-PCR for *Rv1403c* and *hupB*. qRT-PCR data showing the expression of the genes were normalized with 16S rRNA and compared with untreated control. The data shown are the result of two independent experiments. Results are expressed as mean ± SD. ‘***’ $p<0.001$
